# DNA Double-Strand Break Response and Repair Gene Polymorphisms May Influence Therapy Results and Prognosis in Head and Neck Cancer Patients

**DOI:** 10.3390/cancers15204972

**Published:** 2023-10-13

**Authors:** Dorota Butkiewicz, Małgorzata Krześniak, Agnieszka Gdowicz-Kłosok, Krzysztof Składowski, Tomasz Rutkowski

**Affiliations:** 1 Center for Translational Research and Molecular Biology of Cancer, Maria Skłodowska-Curie National Research Institute of Oncology, Gliwice Branch, 44-102 Gliwice, Poland; 2 I Radiation and Clinical Oncology Department, Maria Skłodowska-Curie National Research Institute of Oncology, Gliwice Branch, 44-102 Gliwice, Poland; 3 Radiotherapy Department, Maria Skłodowska-Curie National Research Institute of Oncology, Gliwice Branch, 44-102 Gliwice, Poland

**Keywords:** DNA repair, genetic polymorphism, head and neck cancer, radiotherapy, survival, CHEK1, MRE11, XRCC5, XRCC6, RAD51, LIG4, ATM, TP53, NBN

## Abstract

**Simple Summary:**

Head and neck cancer (HNC) is characterized by radio- and chemoresistance contributing to treatment failure and poor prognosis. There is evidence that a common inherited variation related to DNA damage signaling and repair may modulate individual DNA repair capacity and the results of anticancer treatment. This study evaluated the impact of a panel of single-nucleotide polymorphisms in key genes involved in DNA double-strand break response and repair on three clinical endpoints in HNC patients undergoing radiotherapy and cisplatin-based chemoradiotherapy. We identified variants independently associated with therapy outcome and disease progression. Our findings suggest that these germline variants may be potential biomarkers to be used together with conventional clinical factors for better risk stratification in HNC patients receiving DNA-damaging therapy, which may provide a basis for future treatment modifications.

**Abstract:**

Radiotherapy and cisplatin-based chemotherapy belong to the main treatment modalities for head and neck squamous cell carcinoma (HNSCC) and induce cancer cell death by generating DNA damage, including the most severe double-strand breaks (DSBs). Alterations in DSB response and repair genes may affect individual DNA repair capacity and treatment sensitivity, contributing to the therapy resistance and poor prognosis often observed in HNSCC. In this study, we investigated the association of a panel of single-nucleotide polymorphisms (SNPs) in 20 DSB signaling and repair genes with therapy results and prognosis in 505 HNSCC patients treated non-surgically with DNA damage-inducing therapies. In the multivariate analysis, there were a total of 14 variants associated with overall, locoregional recurrence-free or metastasis-free survival. Moreover, we identified 10 of these SNPs as independent predictors of therapy failure and unfavorable prognosis in the whole group or in two treatment subgroups. These were *MRE11* rs2155209, *XRCC5* rs828907, *RAD51* rs1801321, rs12593359, *LIG4* rs1805388, *CHEK1* rs558351, *TP53* rs1042522, *ATM* rs1801516, *XRCC6* rs2267437 and *NBN* rs2735383. Only *CHEK1* rs558351 remained statistically significant after correcting for multiple testing. These results suggest that specific germline variants related to DSB response and repair may be potential genetic modifiers of therapy effects and disease progression in HNSCC treated with radiotherapy and cisplatin-based chemoradiation.

## 1. Introduction

In recent years, an increase in the incidence of head and neck cancer (HNC) has been observed, both in Poland and worldwide [1,2]. HNCs, the majority of which are squamous cell carcinomas (HNSCCs), represent a significant clinical problem as patients often present with advanced disease, and current treatments are associated with high levels of toxicity and resistance, adversely affecting patients’ quality of life and survival rates. Radiation therapy (RT) and cisplatin-based chemotherapy (CT) are essential therapeutic strategies in HNC [2]. Their mechanisms of action are based on the ability to induce various types of DNA damage, both directly and indirectly, the most harmful of which are double-strand breaks (DSBs) and interstrand crosslinks (ICLs). Generated by ionizing radiation (IR) and free radicals, DSBs are substrates for DNA repair proteins belonging to two major pathways: fast, more error-prone non-homologous end joining (NHEJ) and accurate but slower homologous recombination repair (HR). In addition to the repair of DSBs, HR also participates in the repair of ICLs and single-strand gaps [3,4].

DSBs trigger a DNA damage response (DDR) signaling cascade that involves a network of multiple proteins acting as sensors, transducers/mediators or effectors and comprises damage recognition, cell cycle arrest, DNA repair and cell death [5]. The MRE11-RAD50-NBS1/NBN (MRN) protein complex is responsible for initial DSB sensing, signal transduction and response to lesions and functions in both HR and NHEJ [6]. The activation of the ATM-CHEK2 and ATR-CHEK1 checkpoint kinase pathways leads to the phosphorylation of the tumor suppressor protein p53, resulting in the transcription of many downstream target genes that regulate cell cycle, apoptosis, senescence and repair [7]. The ATM and DNA-dependent protein kinase (DNA-PK) promote NHEJ, which represents a dominant pathway for the repair of DSBs in mammalian cells. The active DNA-PK complex consists of a catalytic subunit (DNA-PKcs, also known as PRKDC or XRCC7) and Ku70/Ku80 (alias XRCC6/XRCC5) heterodimer necessary for damage detection. Then, other proteins such as XRCC4 and DNA ligase IV (LIG4) are recruited which allow for the direct ligation of DNA ends [4]. In turn, in the HR pathway, which requires a template strand to repair the break, RAD51 recombinase plays a central role in homology search and strand-exchange events. RAD51 paralogs such as XRCC2 and XRCC3, as well as many other proteins, including BRCA2, RAD52 and RAD54, participate in RAD51 recruitment and the formation and stabilization of the presynaptic filament [3]. Also implicated in DDR is BRCA1 which, via interactions with numerous proteins, affects cell cycle checkpoint activation, transcription regulation and the promotion of HR repair [8].

Impaired DDR mechanisms may lead to genomic instability, as well as malignant transformation, and may be important for anticancer therapy. Rare germline mutations in certain key genes involved in DSB signaling and repair are associated with cancer-prone syndromes and increased sensitivity to IR or chemotherapeutic agents [3,9]. However, numerous data indicate that also common inherited variations in these genes, such as single-nucleotide polymorphisms (SNPs), may modulate DSB repair capacity, susceptibility to cancer and treatment effects [10,11]. Similar to other solid tumors, HNCs are characterized by radio- and chemoresistance, which contribute to poor therapy outcomes and survival. Enhanced DSB repair may be an important mechanism of therapeutic resistance and, consequently, disease progression in many solid cancers [12]. In HNSCC, for example, NBS1/NBN and XRCC5 overexpression was associated with metastasis and locoregional failure after RT [13,14]. In oral cancer, high levels of MRE11 and RAD51 correlated with radiation resistance and poor prognosis [15,16]. Significant inter-individual differences in response to RT and CT are observed in HNC patients which may be due to, among others, host genetic factors such as SNPs. In HNC, SNPs in HR and NHEJ genes have been rarely investigated in the context of treatment efficiency, cancer progression and patient survival. Therefore, the objective of our study was to assess the influence of common SNPs in 20 core genes involved in DSB response and repair on therapy results and prognosis in patients with unresected HNSCC receiving DNA-damaging treatment.

## 2. Materials and Methods

### 2.1. Study Group

The study group comprised 505 Caucasian patients with primary HNSCC, qualified to undergo curative-intent therapy at the Maria Skłodowska-Curie National Research Institute of Oncology in Gliwice. The inclusion criteria were a tumor located in the larynx (LSCC), oropharynx (OPSCC) or hypopharynx (HPSCC), clinical stage T1-4N0-3M0, WHO 0–1 performance status, treatment with radical RT, no surgery for HNC and no previous treatment for other malignancy. The patients received RT alone (*n* = 244, 48%) or combined with cisplatin-based CT (*n* = 261, 52%). The median total radiation dose was 70 Gy (a range of 50–72 Gy). In the combination treatment subgroup (RT + CT), there were 127 (25%) individuals administered induction CT (docetaxel, 75 mg/m^2^, cisplatin, 75 mg/m^2^, and 5-fluorouracil, 750 mg/m^2^, or cisplatin, 100 mg/m^2^, and 5-fluorouracil, 1000 mg/m^2^) and 211 (42%) patients administered concurrent radiochemotherapy (based on cisplatin, 100 mg/m^2^). Details of the treatments and the follow-up of the patients were described previously [17]. The mean age at diagnosis was 59.7 years (a range of 30–87 years), with a median age of 59 years. Of the patients, 362 (72%) had clinical stage III or IV, 398 (79%) were males and 393 (78%) had a history of smoking cigarettes. Clinicopathological data were obtained from the medical records. The demographic and clinical parameters are presented in Table 1. 

### 2.2. SNP Genotyping

Genomic DNA was extracted from frozen peripheral blood using a Genomic Maxi AX kit (A&A Biotechnology, Gdynia, Poland). SNP identification was performed using commercially available Taqman SNP Genotyping Assays (Applied Biosystems, Foster City, CA, USA), following the manufacturer’s standard protocol. Genotyping was repeated in 10% of the randomly selected samples and resulted in 100% concordance.

There were 31 SNPs in the 20 candidate genes investigated in this study (Table S1). We focused on known variants in key genes involved in DSB response and repair that were previously reported in the literature. The selection of variants included SNPs that were functional or possibly functional and/or located in regions likely influencing gene expression or protein levels/function and/or related to cancer, had a minor allele frequency (MAF) ≥ 10% in the European population, a low linkage disequilibrium (LD), determined via a correlation coefficient r^2^ threshold of <0.8 (based on the 1000 Genomes project phase 3 data, EUR population), and a Hardy–Weinberg equilibrium (HWE) *p* > 0.005 (Tables S1 and S2) [18,19,20,21,22,23,24,25,26,27,28,29,30,31,32,33,34,35,36,37,38,39,40,41,42,43]. One SNP (i.e., *XRCC2* rs3218384) that failed to be genotyped using the appropriate Taqman assay was excluded from further analyses.

### 2.3. Statistical Analysis

The study endpoints were overall survival (OS), locoregional recurrence-free survival (LRFS) and metastasis-free survival (MFS). OS was calculated from the date of diagnosis to the date of death from any cause or the last known date alive. LRFS and MFS were calculated from the last day of treatment to the date of clinically detectable relapse (local and/or regional for LRFS and distant for MFS) or the last examination without evidence of disease. Kaplan–Meier plots and a log-rank test were used to compare the survival curves. The association between each SNP and survival was tested under additive, dominant and recessive genetic models, and the best model (i.e., the model with the lowest *p* value) was selected for the analysis. Univariate and multivariate Cox proportional hazards regression was used to estimate the hazard ratios (HRs) and 95% confidence intervals (CIs). All multivariate models were adjusted for potential confounders, including the median age at diagnosis, sex, T stage, N stage, tumor subsite, chemotherapy use, smoking, alcohol use, local and regional relapses (for OS and MFS) and metastasis or second primary cancer (SPC) diagnosed during follow-up (for OS only). A backward stepwise regression was also performed in order to identify independent risk factors for each endpoint. Pearson’s chi-square test was applied to examine the associations between variables and test for deviations from the HWE. Spearman’s correlation was also used. The Bonferroni correction was used to account for multiple testing (with the level of significance set at ≤0.002). However, due to the exploratory nature of this study, uncorrected *p* values were reported, and *p* ≤ 0.05 was considered the threshold for statistical significance. All tests were two-sided, and the analyses were carried out using STATISTICA 13.1 (TIBCO Software Inc., Palo Alto, CA, USA). 

## 3. Results

The genotype distribution is shown in Table S1. The observed MAFs in the group were in line with the data reported for European populations [18]. The median OS was 71.4 months (range 4–161), while the median LRFS and MFS values were not reached. The 5-year OS rate was 54.8%, the 2-year LRFS rate was 73.0% and the 5-year MFS rate was 83.7%. During the follow-up time (median 81.3 months), there were 251 (50%) deaths and 152 (30%) locoregional recurrences, 60 (12%) patients developed distant metastasis and SPCs were diagnosed in 59 (12%) patients. 

**Figure 1:**
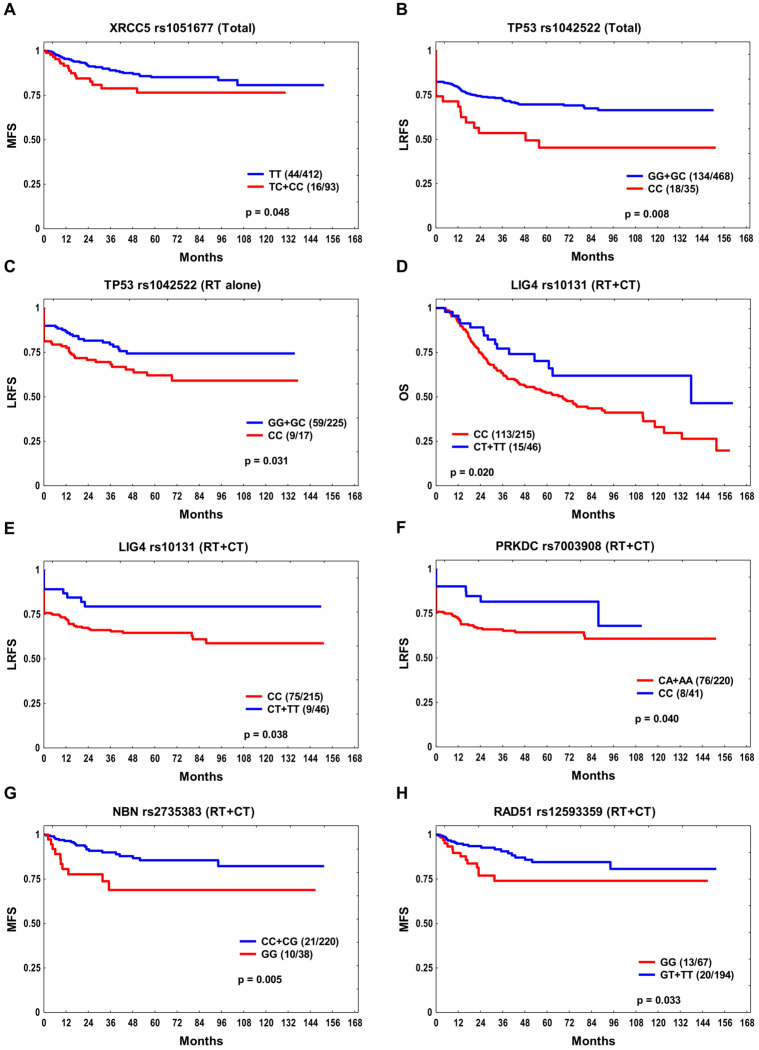
The Kaplan–Meier analysis for the studied SNPs (with *p* ≤ 0.05 only): (**A**) *XRCC5* rs1051677 in relation to metastasis-free survival (MFS) and (**B**) *TP53* rs1042522 in relation to locoregional recurrence-free survival (LRFS) in the whole group; (**C**) *TP53* rs1042522 in relation to LRFS in the RT alone subgroup; (**D**) *LIG4* rs10131 in relation to overall survival (OS); (**E**) *LIG4* rs10131 and (**F**) *PRKDC* rs7003908 in relation to LRFS, and (**G**) *NBN* rs2735383 and (**H**) *RAD51* rs12593359 in relation to MFS in the combination treatment (RT + CT) subgroup. Number of events and *n* are shown in the brackets.

In total, there were six SNPs significantly associated with survival endpoints in the univariate analysis. In the whole group, patients with one or two *XRCC5* rs1051677 C alleles had shorter MFS than TT homozygotes (*p* log-rank 0.048, HR 1.81, 95% CI 1.02–3.20; Figure 1A). The *TP53* rs1042522 CC homozygotes showed reduced LRFS compared to G variant carriers overall (*p* log-rank 0.008, HR 1.88, 95% CI 1.15–3.08; Figure 1B) and in the RT alone subgroup (*p* log-rank 0.031, HR 2.09, 95% CI 1.04–4.21; Figure 1C). In the combination treatment subgroup (RT + CT), the *LIG4* rs10131 CC genotype was associated with unfavorable OS (*p* log-rank 0.020, HR 1.83, 95% CI 1.07–3.14; Figure 1D) and LRFS (*p* log-rank 0.038, HR 1.92, 95% CI 0.96–3.83; Figure 1E). Also, the *PRKDC* rs7003908 A variant conferred a decreased LRFS in this subset (*p* log-rank 0.040, HR 1.96, 95% CI 0.94–4.05; Figure 1F). The *NBN* rs2735383 GG and *RAD51* rs12593359 GG homozygotes treated with RT + CT showed reduced MFS rates (*p* log-rank 0.005, HR 2.85, 95% CI 1.34–6.06, and *p* log-rank 0.033, HR 2.15, 95% CI 1.07–4.32, respectively; Figure 1G,H) compared to other rs2735383 and rs12593359 genotype carriers. None of the associations were statistically significant after correcting for multiple testing.

Multivariate models adjusted for clinicopathological parameters revealed an association between 14 SNPs in 10 genes and the studied endpoints (Table 2). Only four SNPs previously identified in the univariate models (i.e., rs1042522, rs7003908, rs2735383 and rs12593359) were confirmed in this analysis, and *RAD51* rs12593359 was associated with two endpoints (OS and MFS). When all patients were considered, *LIG4* rs1805388 GG, *MRE11* rs2155209 TT, *XRCC5* rs828907 T and *RAD51* rs1801321 GG carriers showed an increased risk of death (HR 1.35, *p* = 0.028, HR 1.36, *p* = 0.019, HR 1.41, *p* = 0.022 and HR 1.37, *p* = 0.016, respectively). The *TP53* rs1042522 CC genotype and *ATM* rs1801516 A allele were associated with an elevated risk of locoregional relapse (HR 1.89, *p* = 0.013 and HR 1.48, *p* = 0.029, respectively), whereas individuals with *ATM* rs189037 A, *XRCC6* rs2267437 CC, *NBN* rs1805787 CC and rs1805794 G were at higher risk of distant relapse (HR 2.14, *p* = 0.049, HR 1.89, *p* = 0.023, HR 1.81, *p* = 0.036 and HR 2.00, *p* = 0.020, respectively). In the subgroup given combination treatment (Table 2), *MRE11* rs2155209 TT, *XRCC5* rs828907 T, *RAD51* rs1801321 GG and rs12593359 GG showed an association with an increased risk of death (HR 1.54, *p* = 0.024, HR 1.76, *p* = 0.012, HR 1.58, *p* = 0.018 and HR 1.56, *p* = 0.041, respectively), while there was only one variant, *PRKDC* rs7003908 A, that conferred a higher risk of locoregional failure (HR 2.14, *p* = 0.045). The *XRCC6* rs2267437 CC, *RAD51* rs12593359 GG, *NBN* rs1805794 GG and rs2735383 GG genotypes were associated with an elevated risk of distant relapse after RT + CT (HR 2.44, *p* = 0.022, HR 2.88, *p* = 0.004, HR 3.12, *p* = 0.017 and HR 3.22, *p* = 0.005, respectively). In patients treated with RT alone, two SNPs showed an association with the studied outcomes. The carriers of *CHEK1* rs558351 TT genotype were at a 2.5-fold higher risk of death compared to variant C carriers (HR 2.54, *p* = 2 × 10^−5^), and individuals with the *TP53* rs1042522 CC genotype had a more than two-fold increased risk of locoregional recurrence (HR 2.16, *p* = 0.047; Table 2). After the adjustment for multiple comparisons, only the effect of *CHEK1* rs558351 on OS remained statistically significant.

By performing a stepwise selection procedure, 10 of the above SNPs were identified as independent predictors of OS, LRFS or MFS (Table 3). In the whole group, *MRE11* rs2155209 TT, *XRCC5* rs828907 T, *RAD51* rs1801321 GG, *LIG4* rs1805388 GG, N > 0 and local and regional relapse, as well as metastasis or an SPC, were independent risk factors for an unfavorable OS. The *TP53* rs1042522 CC and *ATM* rs1801516 A, together with T3–4, N > 0 and non-oropharyngeal tumor localization, were independent indicators of poor LRFS, while the *XRCC6* rs2267437 CC genotype, HPSCC and regional recurrence after treatment had independent negative effects on MFS. In patients who received the combination therapy (RT + CT), *MRE11* rs2155209 TT, *XRCC5* rs828907 T, *RAD51* rs1801321 GG, alcohol consumption, local and regional failure, and metastasis or an SPC were independent indicators of poor OS, whereas *RAD51* rs12593359 GG, *NBN* rs2735383 GG, non-OPSCC and regional relapse independently predicted shorter MFS. There were no independent predictors of LRFS in the RT + CT subgroup. In the RT alone subgroup, *CHEK1* rs558351 TT, N > 0, local recurrence after treatment and metastasis or an SPC were found to be independent risk factors for inferior OS. The *TP53* rs1042522 CC genotype, T3–4, N > 0 and non-OPSCC were independently associated with reduced LRFS, while regional recurrence was the only independent risk factor for MFS in these patients.

## 4. Discussion

There are many indications that common germline variants in genes involved in DSB response and repair may modify the efficiency of these processes and thus be of great importance for cancer susceptibility as well as the success of anticancer treatments based on DNA damage induction, such as DSBs. However, there are few data regarding the role of SNPs in these genes as predictors of therapeutic response and patient survival in HNC since previous reports mainly explored radiation-induced toxicity or cancer risk. In this study, we hypothesized that these SNPs could modulate individual sensitivity to radiation and chemoradiation by altering the levels and activity of the encoded proteins, thus resulting in variable treatment efficacy and prognosis in HNSCC. Using a multivariate analysis, we identified 14 SNPs associated with survival outcomes in HNSCC patients treated non-surgically with RT alone or with combination therapy, 10 of which were independent indicators of unfavorable OS, LRFS or MFS in the final models. One variant, i.e., *CHEK1* rs558351, survived adjusting for multiple comparisons. 

In our report, *MRE11* rs2155209 TT, *XRCC5* rs828907 T, *LIG4* rs1805388 GG and *RAD51* rs1801321 GG independently predicted poor OS in the entire cohort, and rs2155209 TT, rs828907 T and rs1801321 GG were also independent risk factors for OS in the combination treatment subgroup. Possessing endonuclease and exonuclease activities, MRE11 is the core of the MRN complex and is essential in early DSB recognition and signaling, cell cycle checkpoint regulation, telomere maintenance, DNA recombination, meiosis, and immune response to viral infections [6]. The data show that MRE11 may be a biomarker of response to RT and its altered expression correlates with radiosensitivity in cancer. In oral cancer, high MRE11 levels were associated with advanced stage, progression and metastasis and radio- and chemoresistance, as well as reduced OS [15]. The rs2155209 T>C SNP in the 3′ untranslated region (UTR) may influence microRNA (miRNA) binding and gene expression. It was predicted to be within the miR-584, miR-744, miR-1296 and miR-296–5p binding sites, and the C allele showed reduced activity in the reporter gene assay [19,44]. The C variant was also shown to increase the risk of bladder and breast cancers [45,46] while reducing the risk of colorectal cancer [19]. To date, no study has examined this SNP in HNC, and its prognostic significance has only been found in colorectal cancer [19]. In our HNSCC patients, it was the TT genotype that conferred an increased risk of death, which is in line with the observation by Naccarati et al. [19] that the T variant resulted in higher *MRE11* expression, presumably due to less effective interactions with miRNAs. 

Similar to MRE11, XRCC5 (Ku80) functions in a protein complex and, together with XRCC6 (Ku70), participates in DSB recognition in NHEJ. In addition, the XRCC6/XRCC5 dimer is involved in DDR, V(D)J recombination and telomere maintenance. In HNC, XRCC5 overexpression correlated with radioresistance, locoregional failure and mortality [14,47]. The *XRCC5* rs828907 -1401G>T is located in the promoter region, what may affect gene expression, and has been linked to increased cancer susceptibility in the Asian population. In HNC, the T allele has been identified as a risk factor for oral cancer [48] and for RT-induced subcutaneous fibrosis in OPSCC [49]. In our HNSCC patients, the T variant contributed to a shorter OS; however, no studies to date have addressed the functional significance of this SNP or its role in cancer prognosis. Thus, our work is the first to draw attention to its possible impact on cancer survival and may be of additional clinical relevance in HNC as the data indicate a potential predictive role of XRCC5 in immunotherapy combined with RT [50]. 

The ATP-dependent DNA ligase 4 is required for NHEJ and V(D)J recombination, and LIG4 deficiency syndrome, caused by rare *LIG4* gene mutations, is manifested via increased radiosensitivity, neurological abnormalities, immunodeficiency and predisposition to cancer [9]. In oral cancer, a high level of LIG4 expression has been found to predict an unfavorable outcome [51]. In our HNSCC group, the *LIG4* rs1805388 GG genotype was independently associated with a shorter OS. The rs1805388 G>A causes a Thr to Ile exchange at codon 9 (T9I), and the Ile (A) variant has been shown to impair the ligation and adenylation activity of the protein [43]. The Ile variant was also found to be associated with high chromosomal instability in lung cancer [52] and increased DNA damage levels after occupational exposure to lead [53]. This would suggest better repair in the G (Thr) carriers, thus supporting our findings and the hypothesis that more efficient DNA damage removal may lead to worse therapy outcomes. However, other authors have reported an increased radioresistance in healthy individuals [54] and higher mRNA expression in bone marrow [55] in A allele carriers. Similarly, data on rs1805388 in relation to cancer risk and prognosis are inconsistent. In small HNC studies, it was not associated with RT toxicity, while the A allele was protective against cancer [56,57]. The effect on survival has only been studied in lung cancer and, contrary to our observations, the A allele was a risk factor for progression and poor response to platinum-based CT [58]. 

The RAD51 recombinase is a critical player in the HR pathway that interacts with many other key proteins, including BRCA1, BRCA2, CHEK1 and p53. It is upregulated in various cancers, which has been linked to resistance to RT and anticancer drugs [59]. RAD51 overexpression has also been shown to lead to the transcriptional activation of pro-metastatic genes and the promotion of cancer progression [60]. Elevated RAD51 levels were found in oral cancer, especially in patients with lymph node metastases, and indicated a poor prognosis [16]. The *RAD51* rs1801321 -61G>T (alias -172G>T) in the 5′UTR was demonstrated to enhance promoter activity [24]. Similarly, an in silico analysis showed that this SNP is located in the P300/CBP transcription factor binding site, and the T variant may increase *RAD51* expression [61]. In addition, healthy TT homozygotes had lower levels of IR-induced chromatid breaks [61], while in GG carriers, higher levels of DNA damage, detected via a comet assay, were observed [62]. With respect to cancer risk, the T allele showed a protective effect in HNC but had no impact in other cancers [61,63]. The above data could suggest that this frequently studied functional SNP confers increased DNA damage repair capacity in T variant carriers, which may result in a poorer prognosis. However, in our report, the GG genotype was an independent predictor of reduced OS after the combination treatment and overall. Considering therapy outcomes and prognosis, no association was found between this SNP and RT’s toxic effects in HNC and lung cancer [49,56,64], nor in response to platinum-based CT and OS in gastric cancer [65]. In turn, contrary to our findings, in the only study evaluating the effect of rs1801321 on HNC prognosis, the T allele was associated with poor survival in OPSCC. However, this study group was very small, limited to one subsite, and the result was not confirmed via a multivariate analysis [66]. Thus, further research is warranted to assess the role of *RAD51* rs1801321 in HNC progression and prognosis.

Of note, the strongest association that remained statistically significant in our study after correcting for multiple testing was observed between *CHEK1* rs558351 and OS. We found that the TT genotype was an independent risk factor for poor OS after RT. The CHEK1 kinase is an essential molecule for DDR coordination, cell cycle regulation, cell proliferation and survival [7]. The rs558351 -664C>T is located in the 5′UTR and, according to HaploReg v4.2 [67,68], may disrupt regulatory motifs and protein binding. It has also been predicted to markedly alter the secondary structure of RNA [69]. This may cause a phenotypic effect as such SNPs are able to change the binding affinities of proteins and miRNAs [70]. Very little is known about the role of this variant in cancer and other human diseases. It has only been examined in a large case–control study for colorectal cancer risk in the Chinese population, but no effect was found [69]. Thus, our study is the first to indicate the potential prognostic role of this common SNP in cancer, in particular in RT-treated HNSCC patients. Increased CHEK1 expression has been reported in various cancers, including HNC, and was found to correlate with radioresistance and poor outcome, whereas CHEK1 inhibition has been demonstrated to have radiosensitizing effects [7,71]. One could speculate that the T variant, possibly conferring a higher level of CHEK1 activity, may result in limited sensitivity to RT in TT homozygotes and thus in reduced OS. Our data suggest that this SNP deserves further attention, and efforts are needed to explore its functional relevance, role in solid cancer progression and treatment response. Our observation also takes on more importance since CHEK1 is considered a promising therapeutic target in HNC [7,72].

This study identified two SNPs independently related to a high risk of locoregional failure. We demonstrated that the *TP53* rs1042522 CC homozygotes did not benefit from treatment with either RT alone or overall. This well-known, non-synonymous SNP (nsSNP) occurring in the proline-rich domain and resulting in G>C transversion at codon 72 (R72P, Arg72Pro) in exon 4 has been extensively functionally investigated, and significant biochemical and biological differences between the variants have been found. The 72Arg variant was shown to induce apoptosis more efficiently, while the 72Pro was more effective in G1 cell cycle arrest and activating DNA repair [33,34]. The rs1042522 has been linked to the risk of various cancers and other diseases, as well as to aging. Data on HNC do not clearly confirm the relationship between rs1042522 and the development of this cancer, but some suggest that the 72Pro may be a susceptibility allele [73,74]. In HNC, studies on the predictive and prognostic value of this SNP are very scarce. Consistent with our results, the 72Pro allele was also associated with poor prognosis in early-stage HNSCC treated with RT [75] and in breast cancer [76]. The higher DNA repair efficiency in the 72Pro cell lines compared to the 72Arg cells [77] and the lower apoptotic activity of the variant may result in a worse therapy response, although it has been reported that the impact of rs1042522 on the clinical outcome may depend on the p53 mutation status of the tumor [78]. The second SNP linked to locoregional recurrence in our study was *ATM* rs1801516, causing D1835N substitution in exon 37. This nsSNP may alter the exonic splicing enhancer and regulatory motifs, but its functional consequences are unclear [32]. It has been primarily examined in the context of radiosensitivity. The 1835Asn variant was found to be related to increased RT-induced normal tissue toxicity in a meta-analysis including breast and prostate cancers [79], as well as in a small nasopharyngeal cancer study [80]. There are very few data on the prognostic impact of this SNP in cancer. In our HNSCC cohort, the A (1835Asn) allele was an independent indicator of poor LRFS, confirming previous observations showing the association of the AA genotype with progression in colorectal cancer [81]. 

In this report, the *XRCC6* rs2267437 CC, *NBN* rs2735383 GG and *RAD51* rs12593359 GG genotypes independently predicted an increased risk of distant failure. The rs2267437 -61C>G (alias -1310C>G) functional SNP in the *XRCC6* promoter region affects the binding of transcription factors and gene expression [39,40]. In line with our results, healthy carriers of the C allele had lower rates of DSB induction, which may suggest lower radiosensitivity [54]. The C variant was also shown to cause higher promoter activity and increased mRNA levels [40]. Although no correlation was found in HNC between this SNP and cancer susceptibility or RT toxicity, meta-analyses demonstrated that the G allele may be a risk factor in other cancers [56,57,82]. Two previous studies on HNC and renal cancer also showed no association with prognosis [40,83]. Therefore, our work is probably the first to indicate that rs2267437 contributes to the progression of HNSCC. Furthermore, we reported that the rs2735383 GG and rs12593359 GG homozygotes were more than twice as likely to develop distant relapse after combination therapy. Both SNPs are located in 3′UTRs and thus may affect the post-transcriptional regulation of *NBN* and *RAD51* by miRNAs. The rs2735383 541C>G was predicted to be within the binding sites of miR-499-5p, miR-508-3p, miR-629 and miR-509-5p [23,44]. The C allele was shown to decrease the gene expression and alter the binding of miR-509-5p in colorectal cancer [23], as well as miR-629 binding in lung cancer cells [22]. Also, the mRNA levels in CC genotype carriers were found to be lower in colorectal, lung and laryngeal cancer tissues [22,23,84]. Thus, it could be assumed that the C variant is associated with less efficient DNA repair, especially as CC homozygotes had higher levels of cancer risk, including LSCC [84,85], and more chromatid breaks in lymphocytes [22]. Although a few previous reports on the impact of *NBN* rs2735383 on survival demonstrated no correlation in bladder, breast and oropharyngeal cancers [44,86], the existing data, together with our findings, may suggest that the GG genotype is a marker for effective DSB early response and repair, resulting in lower sensitivity to DNA-damaging treatment. In turn, the functional *RAD51* rs12593359 T>G was proposed to affect miR-129-3p binding, and the GG genotype correlated with lower mRNA levels in lymphoblastoid cell lines and fibroblasts, as well as with better OS in hepatocellular carcinoma [87,88]. Conversely, in our RT + CT subgroup, the GG carriers were at a higher risk of metastasis. In HNC, no prior research addressed the prognostic role of rs12593359, and the only case–control study showed no association with HNSCC risk [89]. Given the correlation of RAD51 overexpression with treatment resistance and an aggressive phenotype in many cancers, as well as the therapeutic potential of RAD51 inhibitors [59], subsequent studies on *RAD51* genetic variants may yield clinically valuable results. 

To summarize, our data show that *MRE11* rs2155209 TT, *XRCC5* rs828907 T, *RAD51* rs1801321 GG, rs12593359 GG, *LIG4* rs1805388 GG, *CHEK1* rs558351 TT, *TP53* rs1042522 CC, *ATM* rs1801516 A, *XRCC6* rs2267437 CC and *NBN* rs2735383 GG independently predict an unfavorable clinical outcome in HNSCC patients receiving DNA-damaging therapy. In the stratified analysis, these SNPs were specific risk modifiers with effects that varied by treatment. This is also the first report demonstrating the association of *MRE11* rs2155209, *XRCC5* rs828907, *LIG4* rs1805388, *ATM* rs1801516 and *RAD51* rs12593359 with survival in HNSCC, as well as the first to indicate that *CHEK1* rs558351 may play a role in cancer disease. Nevertheless, several limitations of this study should be noted. First, although our HNSCC group is one of the largest studied in this context, the still relatively small sample size, especially in the stratified analysis, affected the statistical power and reliability of the results. Second, the study group included only Caucasian patients; therefore the observed associations may not be generalizable to other populations and ethnic groups. Third, our candidate gene study evaluated a limited number of SNPs, as well as clinical and demographic features, without taking into account the impact of many other factors, including numerous genetic alterations, on treatment outcomes, progression and prognosis in HNC. We also did not explore the potential effects of pathways or haplotypes or the possibility of gene–gene interactions, which reduced the strength of the presented data. Another potential limitation was the difficulty in interpreting our findings due to the scarcity and frequent inconsistencies of functional studies and/or previous research on the topic. Given all of the above, the results of our study should be interpreted with caution. Well-designed, larger studies are also needed to confirm reported associations, as are more detailed functional studies.

## 5. Conclusions

One way to help improve the survival of HNC patients is to better understand the host genetic factors underlying the mechanisms that modulate treatment sensitivity. Our observations may suggest that common germline variants in crucial DSB response and repair genes have the potential to constitute predictive and prognostic biomarkers for use alongside conventional clinical factors in HNSCC treated using DNA damage-inducing therapies. They may also be of broader importance for anticancer therapy, as DDR inhibition or deficiency increases the efficacy of immunotherapy.

## Figures and Tables

**Table 1:** The characteristics of the study population.

Parameter	Total *n* = 505	RT + CT *n* = 261	RT Alone *n* = 244
Age at diagnosis (median)			
<59 years	235 (47%)	153 (59%)	82 (34%)
≥59 years	270 (53%)	108 (41%)	162 (66%)
Sex			
Male	398 (79%)	53 (20%)	54 (22%)
Female	107 (21%)	208 (80%)	190 (78%)
Tumor site			
Oropharynx	212 (42%)	147 (56%)	65 (27%)
Hypopharynx	63 (12%)	47 (18%)	16 (6%)
Larynx	230 (46%)	67 (26%)	163 (67%)
T stage			
1–2	252 (50%)	81 (31%)	171 (70%)
3–4	253 (50%)	180 (69%)	73 (30%)
N stage			
0	207 (41%)	40 (15%)	167 (68%)
1–3	298 (59%)	221 (85%)	77 (32%)
Smoking status			
Never	112 (22%)	61 (23%)	51 (21%)
Ever	393 (78%)	200 (77%)	193 (79%)
Alcohol consumption ^a^			
Never	124 (25%)	65 (25%)	59 (24%)
Ever	378 (75%)	194 (75%)	184 (76%)

RT, radiotherapy; CT, chemotherapy; RT + CT, combination treatment. ^a^ No data for three patients.

**Table 2:** The associations between SNPs and the studied endpoints in the multivariate analysis (only SNPs with *p* ≤ 0.05 are shown).

Gene	SNP	Genotype	Total			RT + CT			RT Alone		
			Events/*n*	HR (95% CI)	*p*	Events/*n*	HR (95% CI)	*p*	Events/*n*	HR (95% CI)	*p*
OS											
*LIG4*	rs1805388	GG	165/322	1.35 (1.03–1.77)	0.028	-	-	-	-	-	-
*MRE11*	rs2155209	TT	131/245	1.36 (1.05–1.75)	0.019	72/134	1.54 (1.06–2.23)	0.024	-	-	-
*XRCC5*	rs828907	GT + TT	182/354	1.41 (1.05–1.88)	0.022	98/189	1.76 (1.13–2.72)	0.012	-	-	-
*RAD51*	rs1801321	GG	105/203	1.37 (1.06–1.77)	0.016	55/109	1.58 (1.08–2.30)	0.018	-	-	-
*RAD51*	rs12593359	GG	-	-	-	33/67	1.56 (1.02–2.38)	0.041	-	-	-
*CHEK1*	rs558351	TT	-	-	-	-	-	-	38/67	2.54 (1.66–3.90)	2 × 10^−5^
LRFS											
*TP53*	rs1042522	CC	18/35	1.89 (1.14–3.12)	0.013	-	-	-	9/17	2.16 (1.01–4.62)	0.047
*ATM*	rs1801516	GA + AA	47/133	1.48 (1.04–2.12)	0.029	-	-	-	-	-	-
*PRKDC*	rs7003908	CA + AA	-	-	-	76/220	2.14 (1.02–4.50)	0.045	-	-	-
MFS											
*ATM*	rs189037	GA + AA	51/396	2.14 (1.00–4.57)	0.049	-	-	-	-	-	-
*NBN*	rs1805787	CC	38/281	1.81 (1.04–3.16)	0.036	-	-	-	-	-	-
*XRCC6*	rs2267437	CC	22/128	1.89 (1.09–3.26)	0.023	13/68	2.44 (1.14–5.26)	0.022	-	-	-
*NBN*	rs1805794	CG + GG	41/306	2.00 (1.12–3.58)	0.020	-	-	-	-	-	-
*NBN*	rs1805794	GG	-	-	-	7/32	3.12 (1.22–7.95)	0.017	-	-	-
*NBN*	rs2735383	GG	-	-	-	10/38	3.22 (1.42–7.32)	0.005	-	-	-
*RAD51*	rs12593359	GG	-	-	-	13/67	2.88 (1.39–5.96)	0.004	-	-	-

RT, radiotherapy; RT + CT, combination treatment; HR, hazard ratio; CI, confidence interval; OS, overall survival; LRFS, locoregional recurrence-free survival; MFS, metastasis-free survival.

**Table 3:** The final models for OS, LRFS and MFS (stepwise multiple regression analysis).

Endpoint	Variables	HR (95% CI)	*p*
**Total**			
OS	N > 0	1.34 (1.02–1.76)	0.038
	Local recurrence	4.43 (3.34–5.89)	<1 × 10^−6^
	Regional recurrence	1.85 (1.32–2.58)	0.0003
	Metastasis/SPC	1.97 (1.50–2.59)	1 × 10^−6^
	*MRE11* rs2155209 TT	1.29 (1.00–1.65)	0.048
	*XRCC5* rs828907 GT + TT	1.36 (1.02–1.81)	0.038
	*LIG4* rs1805388 GG	1.33 (1.01–1.74)	0.040
	*RAD51* rs1801321 GG	1.32 (1.02–1.70)	0.037
LRFS	T3–4	1.78 (1.25–2.54)	0.001
	N > 0	1.65 (1.12–2.41)	0.011
	Non-OPSCC	1.71 (1.20–2.44)	0.003
	*TP53* rs1042522 CC	1.90 (1.16–3.12)	0.011
	*ATM* rs1801516 GA + AA	1.47 (1.04–2.09)	0.030
MFS	HPSCC	3.06 (1.64–5.70)	0.0004
	Regional recurrence	5.14 (2.94–9.02)	<1 × 10^−6^
	*XRCC6* rs2267437 CC	1.78 (1.05–3.03)	0.032
**RT + CT**			
OS	Alcohol: ever	2.12 (1.31–3.43)	0.002
	Local recurrence	5.36 (3.51–8.20)	<1 × 10^−6^
	Regional recurrence	1.85 (1.22–2.81)	0.004
	Metastasis/SPC	2.37 (1.59–3.52)	2 × 10^−5^
	*MRE11* rs2155209 TT	1.51 (1.05–2.18)	0.026
	*XRCC5* rs828907 GT + TT	1.67 (1.08–2.56)	0.020
	*RAD51* rs1801321 GG	1.49 (1.03–2.16)	0.034
MFS	Non-OPSCC	2.13 (1.03–4.42)	0.042
	Regional recurrence	5.43 (2.51–11.75)	2 × 10^−5^
	*NBN* rs2735383 GG	2.74 (1.28–5.87)	0.010
	*RAD51* rs12593359 GG	2.31 (1.10–4.86)	0.027
**RT Alone**			
OS	N > 0	2.27 (1.55–3.33)	3 × 10^−5^
	Local recurrence	4.27 (2.88–6.31)	<1 × 10^−6^
	Metastasis/SPC	2.45 (1.66–3.60)	6 × 10^−6^
	*CHEK1* rs558351 TT	2.47 (1.63–3.77)	2 × 10^−5^
LRFS	T3–4	3.12 (1.87–5.21)	1 × 10^−5^
	N > 0	1.90 (1.11–3.26)	0.020
	Non-OPSCC	2.01 (1.09–3.68)	0.025
	*TP53* rs1042522 CC	2.15 (1.05–4.41)	0.036
MFS	Regional recurrence	6.54 (2.81–15.24)	1 × 10^−5^

RT, radiotherapy; RT + CT, combination treatment; HR, hazard ratio; CI, confidence interval; OS, overall survival; LRFS, locoregional recurrence-free survival; MFS, metastasis-free survival; HPSCC, hypopharyngeal squamous cell carcinoma; Non-OPSCC, non-oropharyngeal squamous cell carcinoma; SPC, second primary cancer.

## Data Availability

The data presented in this study are available upon reasonable request from the corresponding author.

## Supplementary Materials

The following supporting information can be downloaded at: https://www.mdpi.com/article/10.3390/cancers15204972/s1, Table S1: The genotype frequency in the group, location and functionality of SNPs selected for the study; Table S2: An overview of the most important studies available on the functionality of SNPs selected for the analysis.
