# Supplementary material for: DNA Double-Strand Break Response and Repair Gene Polymorphisms May Influence Therapy Results and Prognosis in Head and Neck Cancer Patients
Source: Cancers (Basel). 2023 Oct 13;15(20):4972. doi: 10.3390/cancers15204972 (PMC10605140; doi:10.3390/cancers15204972)
Supplement: Supplementary file 1 [file cancers-15-04972-s001.zip › cancers-2645185-supplementary.pdf]

Table S1. The genotype frequency in the group, location and functionality of SNPs selected for the study.

Gene	SNP	Chr	Alleles	Region	Genotype distribution [a]	Function [b]	RegDB [c]	EUR MAF [d]	MAF	HWE p val
MRE11	rs2155209	11	T>C	3′UTR	245/209/50	miRNA binding site	1f	0.33	0.31	0.58
	rs499952	11	G>T	intron	196/232/72	---	1f	0.38	0.38	0.80
RAD50	rs2240032	5	C>T	intron	260/197/47	---	1b	0.19	0.29	0.28
NBN	rs1805794	8	C>G; E185Q	exon	197/233/73	NS, splicing (ESE/ESS)	7	0.31	0.38	0.76
	rs2735383	8	C>G	3′UTR	192/231/76	miRNA binding site	7	0.30	0.38	0.64
	rs1805787	8	C>G	intron	281/193/28	---	1f	0.30	0.25	0.49
RAD52	rs1051669	12	C>T	3′UTR	289/191/24	miRNA binding site	1f	0.26	0.24	0.29
RAD54L	rs1048771	1	C>T; A730A	exon	380/118/7	S	1f	0.14	0.13	0.52
RAD51	rs1801321	15	G>T	5′UTR	203/225/77	---	1f	0.42	0.38	0.26
	rs12593359	15	T>G	3′UTR	128/248/129	miRNA binding site	7	0.50	0.50	0.69
XRCC3	rs861539	14	G>A; T241M	exon	200/235/68	NS, splicing (ESE/ESS)	1f	0.39	0.37	0.94
XRCC2	rs3218384	7	C>G	5′UTR	excluded [e]	TFBS	1a	0.20	---	---
BRCA2	rs144848	13	A>C; N372H	exon	280/196/28	NS	6	0.30	0.25	0.41
BRCA1	rs799917	17	G>A; P871L	exon	235/226/42	NS	1f	0.36	0.31	0.23
CHEK1	rs492510	11	A>G	intron	230/219/52	---	1f	0.38	0.32	0.99
	rs558351	11	C>T	5′UTR	130/256/114	TFBS	1b	0.45	0.48	0.58
CHEK2	rs2267130	22	T>C	intron	139/229/130	---	1b	0.48	0.49	0.07
	rs2236142	22	C>G	5′UTR	261/207/30	TFBS	1f	0.32	0.27	0.19
ATM	rs189037	11	A>G	5′UTR	163/233/109	TFBS	1a	0.38	0.45	0.14
	rs1801516	11	G>A; D1853N	exon	371/119/14	NS, splicing (ESE/ESS; abolish domain), possibly damaging	7	0.16	0.15	0.24
ATR	rs2227928	3	G>A; M211T	exon	180/256/67	NS	1f	0.41	0.39	0.11
TP53	rs1042522	17	G>C; R72P	exon	259/209/35	NS	1f	0.29	0.28	0.41
XRCC6	rs2267437	22	C>G	5′UTR	128/255/120	TFBS	1f	0.42	0.49	0.75
	rs132793	22	G>A	3′UTR	363/127/14	---	1f	0.19	0.15	0.48

XRCC5	rs1051677	2	T>C	3'UTR	412/89/4	miRNA binding site	1f	0.11	0.10	0.74
	rs828907	2	G>T	promoter	149/257/97	TFBS	1f	0.44	0.45	0.46
PRKDC	rs7003908	8	A>C	intron	216/207/82	---	4	0.36	0.37	0.01
	rs10109984	8	T>C	intron	186/228/91	---	1f	0.42	0.41	0.15
XRCC4	rs2075685	5	G>T	promoter	153/258/93	TFBS	1f	0.46	0.44	0.39
LIG4	rs1805388	13	G>A; T9I	exon	322/163/19	NS, splicing (ESE/ESS), possibly damaging	7	0.16	0.20	0.77
	rs10131	13	C>T	3'UTR	416/83/5	miRNA binding site	7	0.11	0.09	0.71

SNP, single nucleotide polymorphism; Chr, chromosome number; MAF, minor allele frequency in the studied group; HWE, Hardy-Weinberg equilibrium; S, synonymous; NS, nonsynonymous; TFBS, transcription factor binding site; ESE/ESS; exonic splicing enhancer/exonic splicing silencer; [a] Shown in order of common homozygote/heterozygote/variant homozygote, for some SNPs number of genotypes may be lower than total number of patients due to the lack of genotyping data (amplification problems); [b] Function class according to https://snpinfo.niehs.nih.gov/snpinfo/snpfunc.html; [c] Rank according to RegulomeDB v.2.2, www.regulomedb.org; [d] MAF frequency in European population overall according to www.ensembl.org; [e] SNP excluded from the further analysis due to a call rate < 90%.

Table S2. An overview of the most important studies available on the functionality of SNPs selected for the analysis.

Gene	SNP	Subject of the study	Result	Reference
MRE11	rs2155209	gene expression; luciferase reporter assay	C allele associated with reduced expression	[19]
RAD50	rs2240032	gene expression; luciferase reporter assay; differential transcription factor binding	variant impacted transcriptional activity and transcription factor binding	[20]
NBN	rs1805794	cell migration	185Q (CC) associated with higher migration levels in nasopharyngeal cancer cells	[21]
	rs2735383	gene expression; luciferase reporter assay; mRNA, protein levels in tumor	lower transcription activity found for C variant; CC carriers had lower mRNA and protein levels in tumor tissue	[22]
		gene expression; luciferase reporter assay; mRNA levels in colorectal cancer tissue	C allele shown to decrease the gene expression, and disrupt miRNA binding affinity	[23]
RAD51	rs1801321	gene expression; luciferase reporter assay	promoter activity is enhanced by substituting G at the polymorphic positions for T	[24]

XRCC3	rs861539	apoptosis	lower apoptosis levels associated with Met variant in AA8 CHO cell line	[25]
		homology-directed repair of chromosomal double strand breaks, DSBs	variant protein was functionally active in homologous recombination repair of DSBs	[26]
BRCA1	rs799917	DNA end-joining capacity	higher end-joining capacity for AA genotype	[27]
		mRNA and protein levels; luciferase reporter assay	CC genotype responsible for stronger miR-638 dependent reduction of protein levels in seven cell lines	[28]
		gene expression; luciferase reporter assay	lower gene expression found for C allele in esophageal carcinoma cells; lower mRNA levels in CC carriers in normal and tumor tissues	[29]
CHEK1	rs492510	gene expression; luciferase reporter assay	G allele showed higher transcriptional activity	[30]
ATM	rs189037	gene expression; luciferase reporter assay	TT genotype found to increase *ATM* mRNA expression	[31]
	rs1801516	mRNA expression in lymphoblastoid cell lines	lower gene expression in heterozygous composite allele (5557G>A, IVS38-8T>C; $p = 0.09$)	[32]
TP53	rs1042522	apoptosis, cell cycle distribution	72R was more efficient at inducing apoptosis, while 72P induced higher levels of G1 arrest	[33]
		transcriptional pattern of p53-regulated genes	72R ensured better transcription of apoptotic genes (higher transcription potential)	[34]
		apoptosis	72R showed higher apoptotic potential and ability to localize to the mitochondria	[35]
		transcriptional transactivation; interactions with transcription factors	72Pro was more active transcriptional activator, but no differences in the sequence-specific DNA binding activities; 72Pro exhibited higher ability to bind to TAFII32 and TAFII70	[36]
		protein degradation	72R was more susceptible to HPV E6-mediated degradation	[37]
		gene expression; luciferase reporter assay; proliferation	72R variant increased reporter gene expression and proliferation in pituitary adenoma cells, but reduced expression of *p21* gene	[38]

XRCC6	rs2267437	gene expression; DNA-protein complex formation	G variant found to increase promoter activity and alter DNA-protein complex formation	[39]
		gene expression in normal renal tissues; luciferase reporter assay in various cell lines	lower promoter activity and mRNA levels observed for G allele	[40]
XRCC4	rs2075685	gene expression; luciferase reporter assay	T allele found to increase promoter activity	[41]
		gene expression; luciferase reporter assay; transcription factor binding	G allele shown to increase transcription activity and binding ability of GATA-1; overexpression of mRNA and protein in G carriers	[42]
LIG4	rs1805388	DNA ligase 4 activity	Ile protein showed reduced ligation and adenylation activity (coupled with rs1806389)	[43]

SNP, single nucleotide polymorphism.
